# PREDICTORS OF LIVER FUNCTION AMONG MEXICAN AMERICAN WITH DISABLING ARTHRITIS

**DOI:** 10.1093/geroni/igae098.3458

**Published:** 2024-12-31

**Authors:** Tracie Harrison, Shelley Blozis Villareal, Heather Hunter

**Affiliations:** University of Arkansas for Medical Sciences, Little Rock, Arkansas, United States; University of California Davis, Davis, California, United States; University of Arkansas for Medical Sciences, Fayetteville, Arkansas, United States

## Abstract

In this secondary analysis of data from our recent biobehavioral study of Mexican American men and women with disabling arthritic conditions, we analyzed the relationships between mental health predictors, inflammation and liver enzymes, after controlling for age and sex. Consistent with the theoretical paradigm evaluating the prevalence and impact of diseases of desperation, the relationships between grief, anxiety, depression, inflammation (c-reactive protein, CRP) and liver function (Serum glutamic oxaloacetic transaminase (SGPT)) were analyzed. The 67 men (N=18) and women (N=46) 46-83 years of age (age M=62.4 years) were primarily unemployed (55%), married (43%) and high school graduates (36%). Participants were community residing and recruited through flyers, word of mouth, and known key informants. After IRB approval, surveys and blood samples were collected in homes and then analyzed. Results using Pearson correlations with reliable measures indicated significant relationships between SGPT and CRP (r=.46, P< 0.01), SGPT and anxiety (r=.365, p<.01), SGPT and depression (r=.34, p< 0.02), and SGPT and grief (r=.30, p<.04). Using a regression analysis, after controlling for age and sex, mental health (anxiety, depression, and grief) explained an additional 16% (R2 change =.16, F = 2.4, p =.08) of the variance in liver enzymes. This study was limited to a small sample size. In conclusion, psychological variables may influence physiological outcomes in Mexican Americans aging with severe arthritis, and further research is needed on mental health needs among Mexican Americans aging with disability in the community setting.

